# Causal link between polymyositis and idiopathic pulmonary fibrosis in individuals of European ancestry: A Mendelian randomization and multi-omics study identifying mediators and drug targets

**DOI:** 10.1097/MD.0000000000050168

**Published:** 2026-08-21

**Authors:** Miaomiao Chen, Xia Tong, Ruiying Zhang, Yazheng Zhang, Xin Zhang, Bin Liu

**Affiliations:** a Department of Respiratory and Critical Care Medicine, Tianjin Chest Hospital, Tianjin, China; b Department of Gastroenterology, West China (Airport) Hospital of Sichuan University, Chengdu, China; c Department of Emergency and Critical Care, Tianjin University Chest Hospital, Tianjin, China; d Department of Respiratory and Critical Care Medicine, Chest Hospital, Tianjin University, Tianjin, China.

**Keywords:** circulating proteins, drug targets, idiopathic pulmonary fibrosis, Mendelian randomization, metabolites, molecular docking, multi-omics, polymyositis

## Abstract

Polymyositis (PM), an autoimmune muscle disorder, often leads to interstitial lung disease (ILD), including idiopathic pulmonary fibrosis (IPF). However, causality and mechanisms are unclear. This study aimed to investigate the causal effect of PM on IPF risk, identify potential mediating biomarkers through multi-omics analysis, and explore candidate therapeutic drugs. Two-sample Mendelian randomization (MR) was conducted using publicly available genome-wide association study (GWAS) summary statistics (PM: 244 cases/4,28,965 controls; IPF: 6257 cases/9,47,616 controls). Reverse-causality was evaluated via bidirectional MR. We acknowledge the potential for sample overlap between exposure and outcome datasets when using public GWAS data. To mitigate related biases, we employed robust MR methods, including inverse-variance weighted (IVW) with random effects, MR-Egger, and MR-PRESSO, that are less sensitive to such overlap. Genetic variants with significant missing data or poor imputation quality were excluded as per the quality control standards of the original GWAS. This specific analysis was not preregistered and should be considered exploratory. The analysis proceeded in two sequential phases to identify shared causal mediators: First, MR analyses were conducted with 91 inflammatory proteins, 1400 plasma metabolites, 4907 circulating proteins, and 731 immune cell traits as exposures and IPF as the outcome to identify significant causal biomarkers for IPF. Second, MR analyses were conducted with PM as the exposure and the biomarkers significantly associated with IPF as outcomes to identify those also influenced by PM with consistent effect directions. Identified key proteins underwent functional enrichment analysis. Molecular docking was used to validate interactions between significant mediators and potential drugs. PM significantly increased IPF risk (odds ratio [OR]: 1.06, 95% confidence interval [CI]: 1.02–1.09, *P* < .01), with no significant reverse causal evidence. Five circulating proteins (AMH, CHCHD10, CRABP2, HERC5, HSPA5) and 5 metabolites mediated this association, enriched in endoplasmic reticulum stress and transforming growth factor (TGF-β) signaling. Three drugs (butein, gambogic acid, tauroursodeoxycholic acid) showed strong binding affinity (ΔG < −7.0 kcal/mol). Genetic evidence supports PM causally increasing IPF risk. Multi-omics revealed key mediating biomarkers and underlying biological pathways, while drug target prioritization identified 3 compounds with therapeutic potential. This informs prevention strategies.

## 1. Introduction

Polymyositis (PM) is a rare autoimmune muscle disorder frequently complicated by interstitial lung disease (ILD), which significantly impairs patients’ pulmonary function. Idiopathic pulmonary fibrosis (IPF), as a specific progressive subtype of ILD, is characterized by irreversible interstitial fibrosis and progressive decline in lung function, with poor prognosis and limited treatment options.^[1]^Although both PM and IPF involve immune abnormalities and fibrotic pathological processes, the existence of a direct causal relationship, underlying shared mechanisms, and intervention targets between the 2 remain unclear.

Existing studies indicate that pulmonary involvement in PM patients is closely associated with autoantibody-mediated immune disorders, inflammatory cell infiltration, and abnormal activation of fibroblasts.^[2,3]^ In contrast, the pathogenesis of IPF is thought to be linked to telomere dysfunction, epithelial-mesenchymal transition (EMT), and dysregulated wound repair responses.^[4-6]^ However, it remains unclear whether and how systemic immune abnormalities in PM contribute to the development or progression of pulmonary fibrosis in IPF, and robust causal evidence coupled with biological pathway analysis is lacking. Additionally, association analyses based on traditional observational studies are prone to confounding factors, making it difficult to clarify the causal direction, while integrative multi-omics analysis can provide new insights into revealing the common molecular mechanisms between diseases.

This study aims to validate the causal relationship between PM and IPF through Mendelian randomization (MR) analysis, screen potential mediating factors using multi-omics data (inflammatory proteins, metabolites, circulating proteins, immune cells), and explore targeted intervention strategies via molecular docking technology, with the goal of providing new theoretical evidence for the prevention and treatment of PM-related pulmonary fibrosis.

## 2. Materials and methods

**Figure 1.**
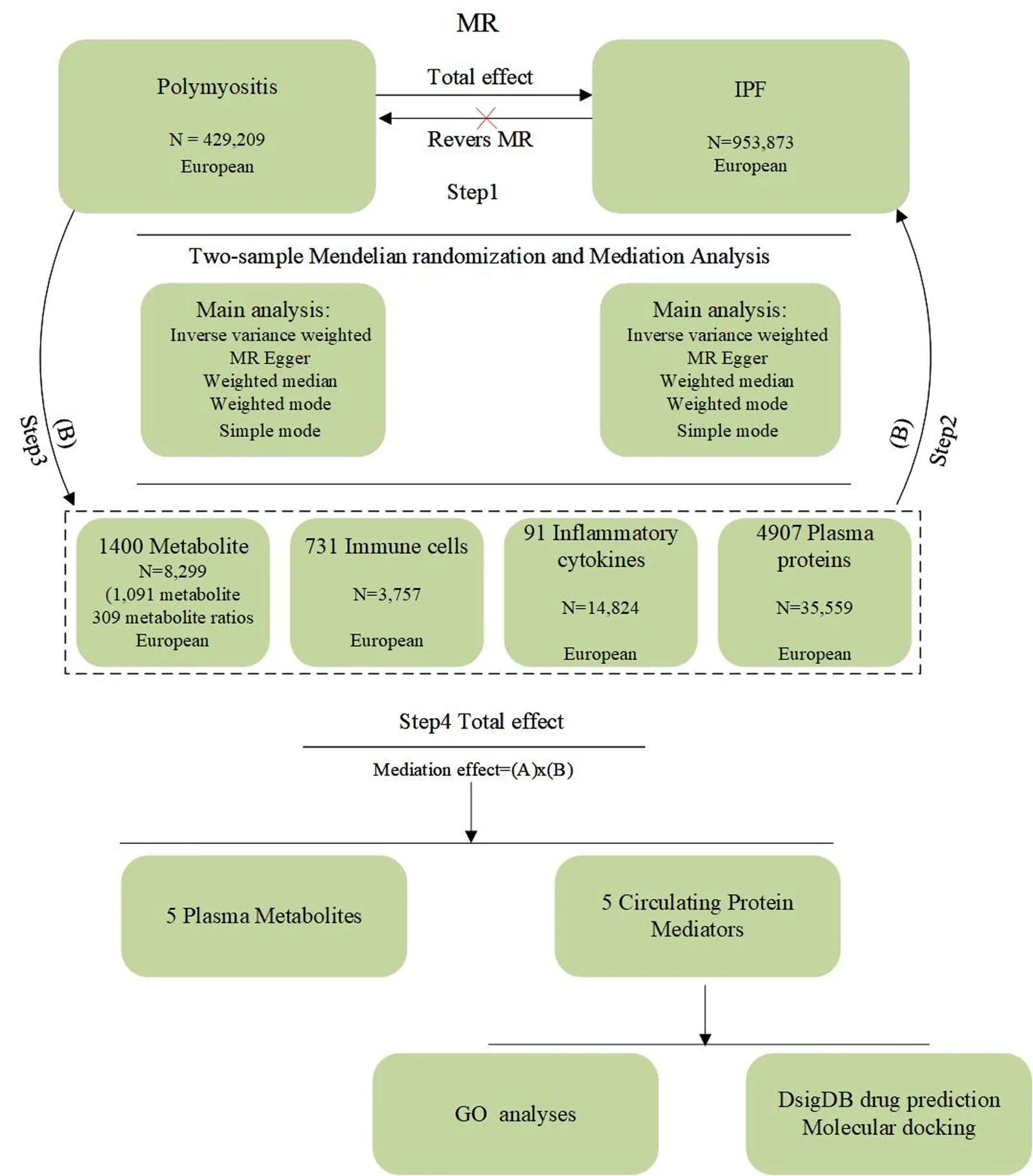
Workflow of MR and multi-omics analysis for PM-IPF causality and drug target discovery. GO = Gene Ontology, IPF = idiopathic pulmonary fibrosis, MR = Mendelian randomization, PM = polymyositis.

The study flow is illustrated in Figure 1.

First, we obtained summary data from published GWAS on IPF, plasma metabolites, immune cells, inflammatory proteins, circulating proteins, and PM. Two-sample MR analysis was used to assess the causal relationship between PM and IPF. Subsequently, 2-step MR analysis was applied to evaluate the mediating roles of plasma metabolites, immune cells, inflammatory proteins, and circulating proteins in the PM-IPF association. Gene Ontology (GO) analyses were performed to clarify the functions of mediating circulating proteins. Finally, candidate drug prediction and molecular docking were used to screen potential therapeutic targets for IPF.

### 2.1. Data sources

*Polymyositis data* (ID: finngen_R10_M13_POLYMYO) were from a large-scale European study with 4,28,965 controls and 244 cases.

*IPF data* (ID: GCST90399720) originated from another large-scale European study, comprising 9,47,616 controls and 6257 cases.^[7]^

### 2.2. Intermediary factors

*91 inflammatory proteins:* The GWAS dataset (registry numbers GCST90274758–GCST90274848) in the GWAS catalogue was derived from the Olink platform, covering GWAS pooled analyses of 91 plasma inflammatory proteins in 14,824 European participants.^[8]^

*1400 metabolites:* Metabolite data from the Canadian Longitudinal Study of Aging (CLSA) cohort included 1091 blood metabolites, 309 metabolite ratios, and ~15.4 million single nucleotide polymorphisms (SNPs) analyzed in 8299 participants.^[9]^

*4907 plasma proteins:* The GWAS dataset of plasma protein levels comes from the largest proteomics study to date. It covers 35,559 participants of European ancestry and measures a total of 4907 proteins.^[10]^

*731 immune cells:* The immune cell dataset comprises GWAS summary data for 731 immune cell types from 3757 European-origin participants.^[11]^

### 2.3. Statistical analysis and selection of instrumental variables

This study employed 2-sample MR analysis to evaluate the potential causal effect of PM on IPF. Primary analyses were conducted using the inverse variance weighted (IVW) method via the TwoSampleMR (v0.5.7) and MR-PRESSO (v1.0) packages in R software (v4.3.1; R Foundation for Statistical Computing, Vienna, Austria). To ensure result robustness, complementary analyses including the weighted median method, weighted plurality method, and MR Egger regression were performed to assess estimate stability and calculate odds ratios (OR) with 95% confidence intervals (CIs). Additionally, reverse MR analysis (IPF as exposure, PM as outcome) was conducted to explore potential reverse causality, with statistical significance set at *P* < .05.

To minimize causal estimation bias, we assessed pleiotropy via the MR-Egger intercept (*P* > .05 = no pleiotropy) and heterogeneity via Cochran Q-test (*P* > .05 = no heterogeneity). Scatter/funnel plots and “leave-one-out” analyses evaluated robustness and detected outlier SNPs influencing overall MR estimates.

Instrumental variables (IVs) were selected following 3 Mendelian randomization assumptions: significant association with exposure, no confounding with the outcome, and exclusive effect on the outcome via exposure. We set a *P*-value threshold of 1 × 10^−5^ and linkage disequilibrium (LD) threshold (*r*^2^ < 0.001) to minimize bias. SNPs associated with outcomes (*P* < .05) were excluded, and pleiotropy was addressed using MR-PRESSO. Only SNPs with *F*-statistic ≥ 10 were retained to ensure IV strength and avoid weak instrument bias.

In the mediated effect analysis, we explored the mechanism of PM on IPF and assessed potential mediating roles of inflammatory proteins, metabolites, circulating proteins, and immune cells. Mediation effects were estimated via a 2-step MR approach: the mediated effect was calculated as β(*A*) × β(*B*), with the direct effect derived from “direct effect = total effect − mediated effect.” The mediating effect ratio was determined by “(mediated effect/ total effect) × 100%,” and 95% CIs were estimated using the delta method to quantify mediator impacts precisely.^[12,13]^ All multi-omics associations were corrected for multiple testing using the false discovery rate (FDR) method, with statistical significance defined as FDR-adjusted *P* < .05.

## 3. Results

### 3.1. Polymyositis increases the risk of idiopathic pulmonary fibrosis

**Figure 2.**
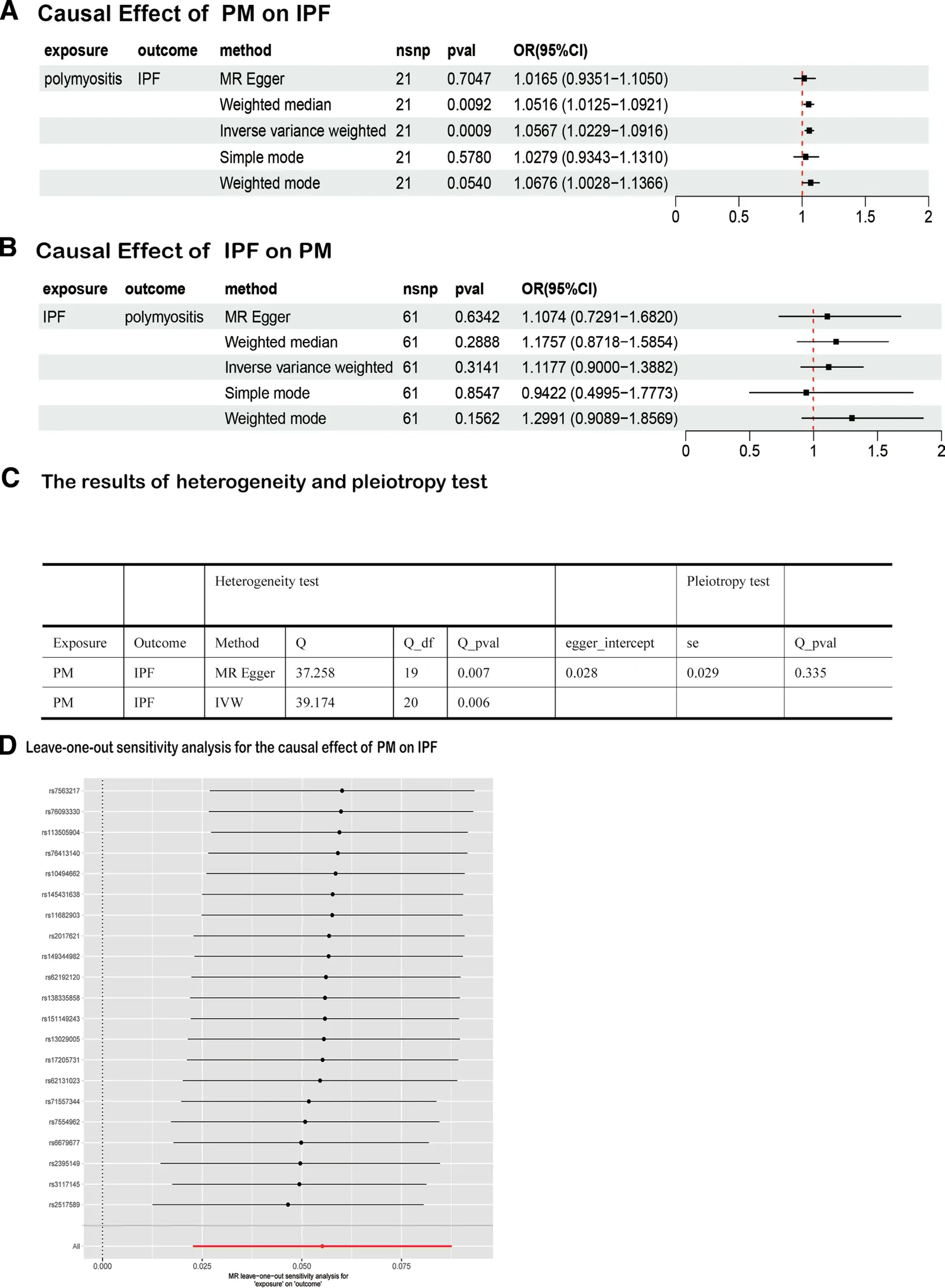
Causal relationship between IPF and PM. (A) MR analysis confirmed that PM is a risk factor for IPF, with the IVW method showing *P* < .05. (B) Reverse MR analysis demonstrated that IPF does not causally influence the risk of PM. (C) Heterogeneity and pleiotropy test results for the causal effect of PM on IPF. (D) Leave-one-out analysis of PM’s causal effect on IPF. CI = confidence interval, IPF = idiopathic pulmonary fibrosis, IVW = inverse variance weighted OR = odds ratio, MR = Mendelian randomization, PM = polymyositis.

We conducted 2-sample Mendelian randomization to identify SNPs associated with both PM and IPF. Results showed PM significantly increased IPF risk (*P* < .05; Fig. 2A), while reverse causality analysis found no evidence of IPF influencing PM onset (*P* > .05; Fig. 2B). Significant heterogeneity was observed (Cochran *Q P* = .006; Fig. 2C); however, no evidence of horizontal pleiotropy was detected (MR-Egger intercept *P* = .335; Fig. 2C), and the leave-one-out sensitivity analysis confirmed that the results were not driven by any single instrumental variable (Fig. 2D). These findings confirm PM as a risk factor for IPF.

### 3.2. Causal mediating biomarkers linking PM exposure to IPF risk

**Figure 3.**
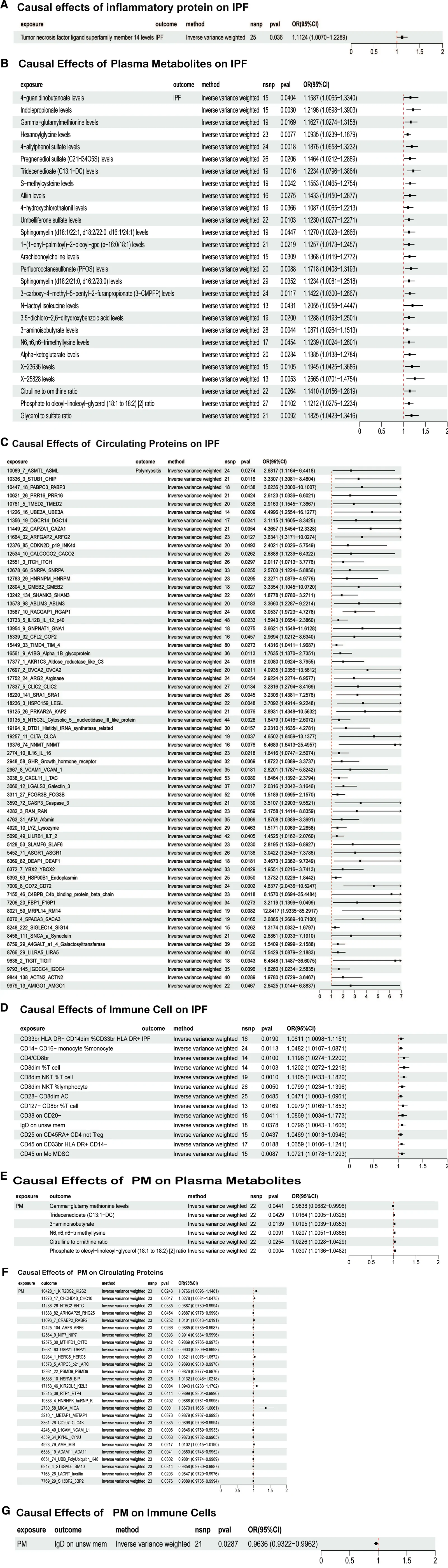
Identification of IPF-associated biomarkers from multi-omics data and assessment of their potential causal relationship with PM. (A) The causal effects of inflammatory proteins on IPF. (B) The causal effects of plasma metabolites on IPF. (C) The causal effects of circulating proteins on IPF. (D) The causal effects of immune cells on IPF. (E) The causal effects of PM on the identified plasma metabolites. (F) The causal effects of PM on the identified circulating proteins. (G) The causal effects of PM on the identified immune cells. CI = confidence interval, IPF = idiopathic pulmonary fibrosis, OR = odds ratio, PM = polymyositis.

We implemented a 2-stage analytical framework to identify shared causal mediators. In the first stage, we performed MR analyses to identify biomarkers with significant causal effects on IPF. We analyzed a total of 91 inflammatory proteins, identifying 1 risk factor associated with IPF (Fig. 3A). No protective factors were detected among these proteins. From the analysis of 1400 plasma metabolites, we identified 27 as risk factors (Fig. 3B). Among the 4907 circulating proteins assessed, 109 were classified as risk factors (Fig. 3C). Additionally, our analysis of immune cells revealed 13 risk factors (Fig. 3D). All findings remained significant after FDR correction (Tables S1, S2, S3 and S4, Supplemental Digital Content 1).

**Figure 4.**
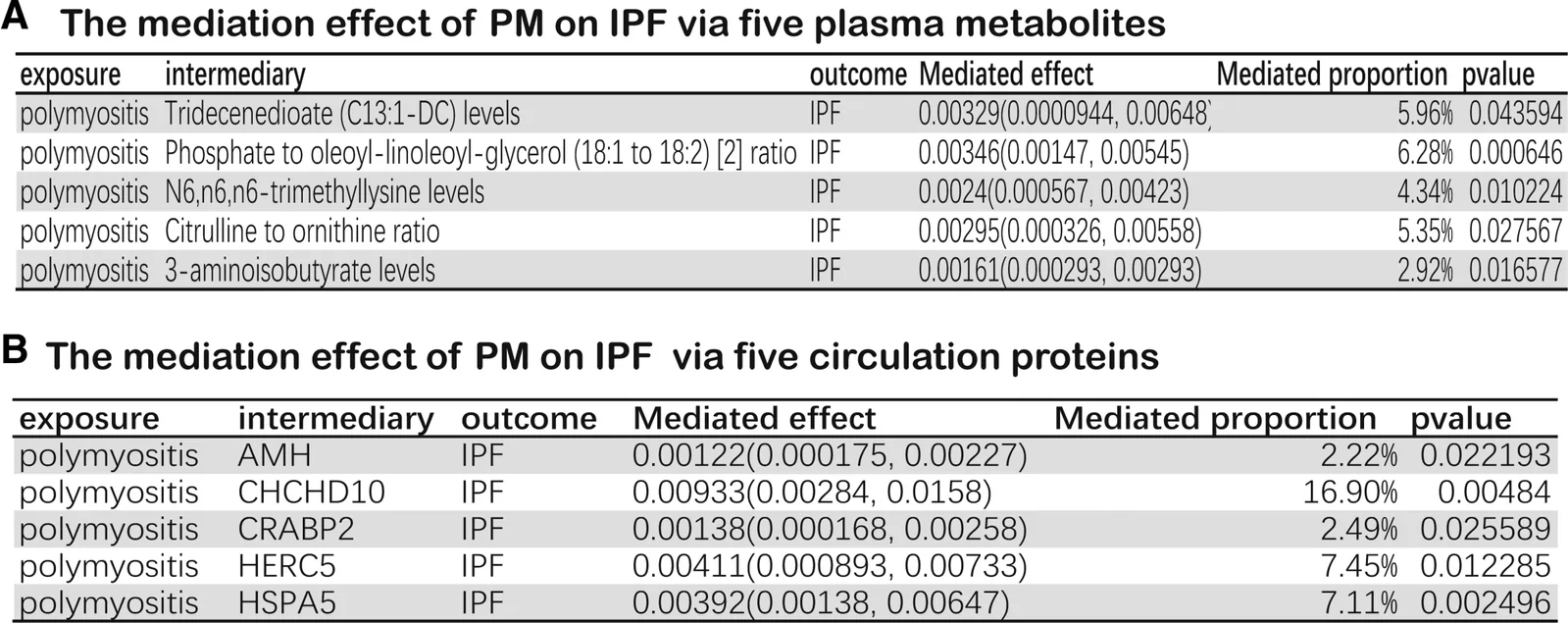
The mediation effect of PM on IPF. (A) The mediation effect of plasma metabolites on IPF. (B) The mediation effect of circulating proteins on IPF. IPF = idiopathic pulmonary fibrosis, PM = polymyositis.

In the second stage, we conducted MR analyses to evaluate whether these IPF-associated biomarkers were, in turn, causally influenced by PM. We performed reverse MR analysis using PM as the exposure on this set of IPF-associated biomarkers (1 inflammatory protein, 27 plasma metabolites, 109 circulating proteins, and 13 immune cells). From this analysis, a subset of biomarkers was identified, comprising 6 plasma metabolites, 27 plasma proteins, and 1 immune cell (Fig. 3E–G). All findings remained significant after FDR correction (Tables S5, S6 and S7, Supplemental Digital Content 2). To further elucidate the causal mechanisms, we performed mediation analyses using PM as the exposure and the above candidate biomarkers as mediators. From this refined subset, a final core set of 5 plasma metabolites and 5 circulating proteins demonstrated consistent and significant mediation effects across overall, indirect, and direct pathways (Fig. 4A, B). Leave-one-out analyses confirmed the robustness of these causal relationships within the 2-sample Mendelian randomization framework of exposure, mediator, and outcome. These findings offer critical insights into the mechanisms by which PM may facilitate the progression of IPF through specific circulating proteins and metabolites.

### 3.3. Functional annotation of mediator circulating proteins via GO analyses

**Figure 5.**
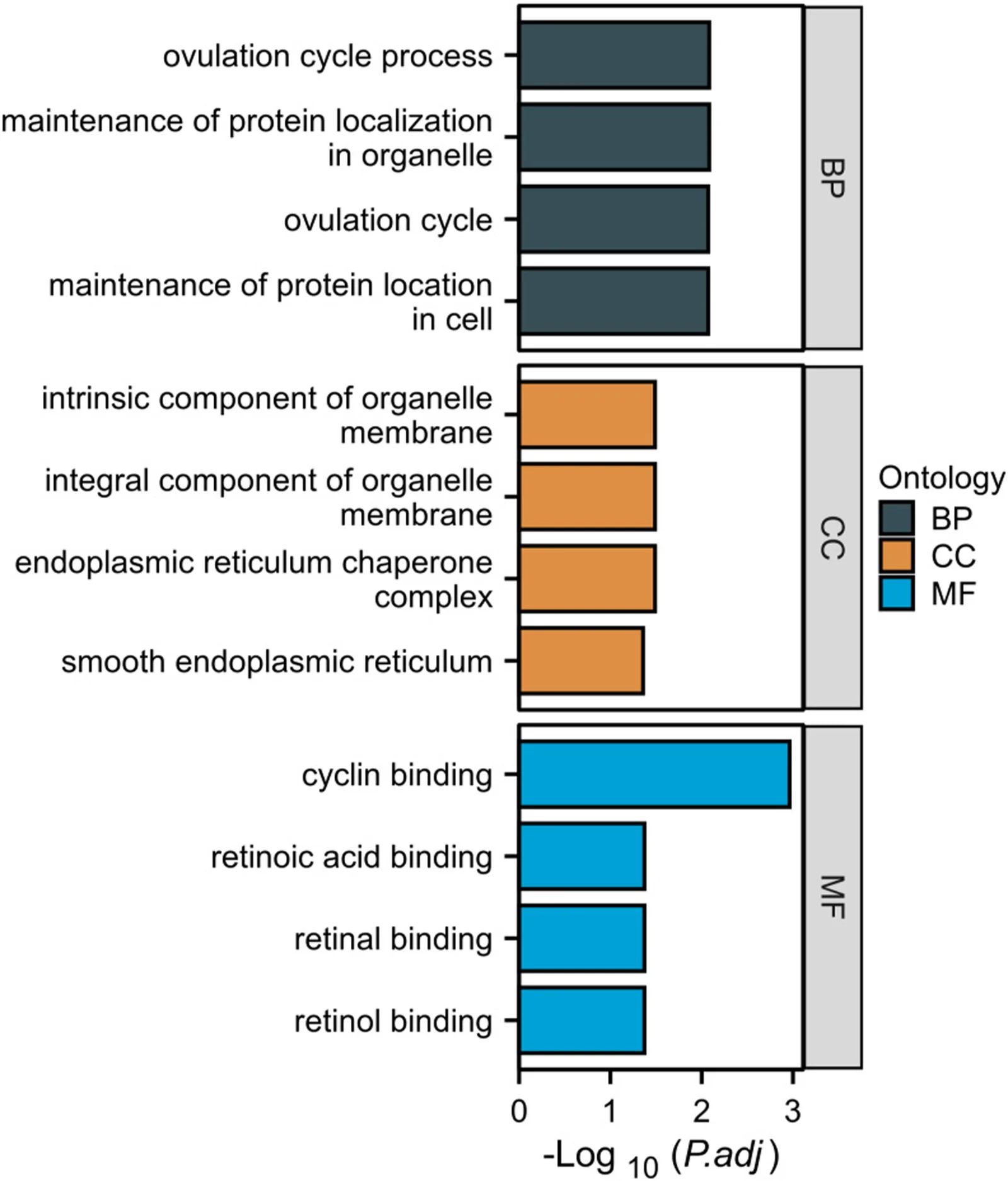
GO analyses reveal biological roles of mediator circulating proteins. GO analysis showed that the 5 mediator circulating proteins are involved in multiple biological roles. BP = biological process, CC = cellular component, GO = Gene Ontology, MF = molecular function.

We performed GO enrichment analyses on the genes encoding these 5 circulating proteins. GO analysis showed these proteins are primarily involved in biological processes such as maintenance of protein localization in organelles and regulation of the ovulation cycle, localize to endoplasmic reticulum chaperone complexes and organelle membrane structures, and exhibit molecular functions including cyclin binding and retinoic acid-related substance binding (Fig. 5).

### 3.4. Candidate drug prediction and molecular docking

**Figure 6.**
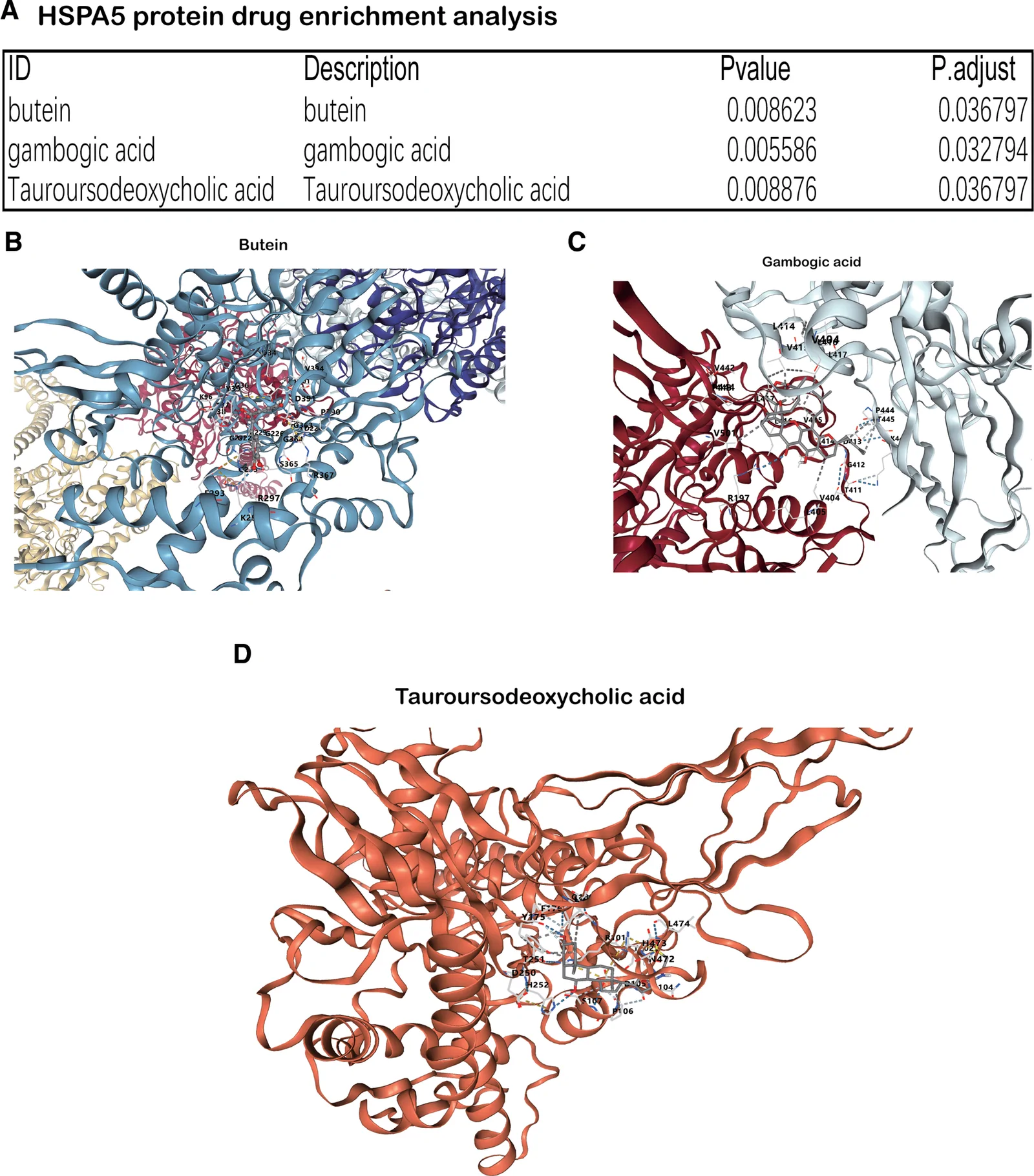
Targeted drug discovery: enrichment analysis and docking validation. (A) HSPA5 protein drug enrichment analysis predicts 3 potentially effective intervention drugs. (B) Molecular docking of butein with HSPA5 protein. (C) Molecular docking of gambogic acid with HSPA5 protein. (D) Molecular docking of tauroursodeoxycholic acid with HSPA5 protein. HSPA5 = heat shock 70 kDa protein 5, CB-Dock2 = a molecular docking platform (software name, used as defined).

In this study, potential intervention drugs were predicted based on the DSigDB database. After screening by adjusted p-values, the CB-Dock2 molecular docking technique was used to evaluate the affinity of candidate drugs for the target and the druggability of the target. The results showed that butein, gambogic acid, and tauroursodeoxycholic acid (TUDCA) exhibited the most significant binding activity with the HSPA5 protein (Vina scores: butein −8.8 kcal/mol; gambogic acid −9.3 kcal/mol; tauroursodeoxycholic acid −8.1 kcal/mol), indicating extremely strong binding stability of the 3 compounds (Fig. 6A–D).

## 4. Discussion

This study employed MR and multi-omics analyses to elucidate the causal relationship between IPF and PM and identify potential therapeutic targets. PM is primarily an autoimmune myopathy driven by systemic inflammation and muscle weakness. Although IPF is traditionally classified as a progressive fibrotic disorder rather than an autoimmune disease, accumulating evidence suggests that autoimmune processes – such as circulating autoantibodies found in approximately one-third of IPF patients^[14^ – may also contribute to its pathogenesis. This overlap provides a biological rationale for investigating a potential causal link. ILD is a well-recognized complication of PM, with reported prevalence rates ranging from 19.9% to 42.6%.^[15,16]^ However, epidemiological associations between PM and ILD (including IPF) identified in prior studies are prone to confounding by factors such as smoking, environmental exposures, and genetic susceptibility. In this study, MR analysis, utilizing genetic variants as instrumental variables, effectively minimized confounding bias and provided robust evidence supporting a causal relationship between PM and IPF.

Therefore, our findings indicate a unidirectional causal effect, where PM significantly increases the risk of IPF (OR: 1.06, 95% CI: 1.02–1.09, *P* < .01), while IPF has no causal impact on PM (as confirmed by bidirectional MR analysis, OR: 1.12, 95% CI: 0.90–1.39, *P* = .31). The apparent disparity in case numbers between IPF (N = 6257) and PM (N = 244) reflects their differential population prevalence in European populations, where IPF demonstrates substantially higher incidence rates (5.1 cases per 1,00,000 persons)^[17]^compared to PM (0.12–1.9 cases per 1,00,000 persons).^[18]^ Crucially, MR methodology relies on genetic instruments that are independent of disease prevalence, ensuring the validity of causal inference regardless of case number differences.

Our metabolomic analysis extends beyond the established link between amino acid/lipid metabolism and IPF^[19]^ by identifying 5 key metabolites and, crucially, establishing their causal roles as mediators driven by PM. This MR study provides causal evidence supporting their role in disease pathogenesis. While previous studies noted elevated levels of structural amino acids (e.g., elastin-derived L-leucine/L-isoleucine in progressive fibrosis^[20]^ and collagen-related proline in IPF^[21,22]^), our study provides a novel pathogenic link by identifying PM as an upstream driver of these metabolic disturbances. Furthermore, we specifically implicate 3 amino acid-related metabolites – 3-aminoisobutyrate, the citrulline-to-ornithine ratio, and N6,N6,N6-trimethyllysine – in IPF pathogenesis. Although these metabolites have been associated with COVID-19 severity, long-COVID, and cardiovascular risk,^[23,24]^ our study is the first to position them within a PM-IPF pathological axis, suggesting a shared mechanism bridging systemic inflammation and fibrosis. Similarly, lipids are crucial for cellular energy storage, structural integrity, and signaling and have been implicated in various respiratory diseases, including cystic fibrosis, asthma, and chronic obstructive pulmonary disease (COPD).^[25-27]^Yan et al identified significant alterations in 62 lipid species through mass spectrometry in IPF patients compared to healthy subjects.^[28]^ Clinical studies have also reported elevated levels of lysophosphatidic acid (LPA) in bronchial lavage (BAL) fluid and exhaled breath in IPF patients.^[29,30]^ Our findings indicate that specific lipid metabolites are not merely biomarkers but causal contributors to IPF progression. This finding provides a mechanistic rationale for targeting lipid metabolism in IPF therapy.

Similarly, our proteomic analysis identifies 5 circulating proteins (AMH, CHCHD10, CRABP2, HERC5, HSPA5) as mediators. While genetic studies have associated circulating proteins with IPF and PM individually,^[31,32]^ our study uniquely demonstrates their causal mediating role between the 2 diseases. We first screened 109 potential IPF-associated circulating proteins, then validated their association with PM and consistent effect directions via 2-step MR – strengthening result specificity. Anti-Müllerian hormone (AMH), a marker of reproductive aging and a member of the TGF-β family, has unclear links to PM. While AMH dysregulation may increase autoimmune risks,^[33]^ and correlates with multiple sclerosis activity,^[34]^ we are the first to demonstrate AMH’s mediating role in PM-induced IPF, suggesting PM may promote IPF fibrosis via AMH-driven TGF-β signaling. Retinol-binding protein CRABP2, dysregulated in lung cell types and linked to reduced lung function,^[35]^ and HERC5 (a HERC family protein involved in neural development, DNA repair, and immunity,^[36]^ with roles in non-small cell lung cancer and hepatocellular carcinoma progression^[37-39]^), are newly implicated in the PM-IPF axis – suggesting they mediate IPF pathogenesis by regulating lung epithelial function and inflammatory responses in PM. CHCHD10, a mitochondrial protein linked to neurodegenerative diseases,^[40]^ is revealed in our study as playing a critical role in the promotion of IPF due to PM. Heat shock protein family A member 5 (HSPA5), also known as glucose-regulated protein 78 (GRP78), has been implicated in various diseases, including cancer and cardiovascular disorders.^[41]^ Notably, as an endoplasmic reticulum (ER) chaperone, HSPA5 (GRP78) emerges as a central node in ER stress, which serves as a hallmark of IPF and is closely linked to epithelial apoptosis and myofibroblast activation.^[42]^ Our analysis indicated that these proteins are not only confirmed as IPF risk factors, but more importantly, identified as key mediators between PM and IPF.

The GO analyses of the identified mediator proteins (AMH, CHCHD10, CRABP2, HERC5, HSPA5) provide mechanistic insights into their roles in PM-driven IPF. GO analysis highlights their involvement in protein localization within organelles (e.g., HSPA5 in ER chaperone complexes), a process critical for maintaining cellular homeostasis. Disruptions in ER protein quality control, mediated by HSPA5, are directly linked to ER stress – a hallmark of IPF that drives epithelial cell apoptosis and myofibroblast activation. The enrichment of AMH in “ovulation cycle regulation” offers a novel mechanistic research direction: as a reproductive aging marker and member of TGF-β superfamily, AMH may modulate immune cells or pulmonary stromal cells via paracrine pathways, thereby influencing pulmonary fibrogenesis.

In conclusion, this study is the first to investigate the risk factors of PM for IPF from multiple perspectives, correlating inflammatory proteins, metabolites, circulating proteins, and immune cells. We identified 5 metabolites and 5 circulating proteins as key mediators of PM-driven IPF development. These discoveries not only provide new insights into the molecular mechanisms underlying the causal association between PM and IPF but also suggest novel directions for future basic research and clinical interventions.

Utilizing the DSigDB database and CB-Dock2 molecular docking technology, we identified butein, gambogic acid, and TUDCA as potential inhibitors of HSPA5, a validated mediator of endoplasmic reticulum stress. These compounds demonstrated excellent binding affinity towards HSPA5. Among them, TUDCA has shown promise in preclinical models by mitigating ER stress in neurodegenerative and metabolic diseases.^[43,44]^ In IPF, TUDCA’s ability to reduce ER stress may alleviate epithelial injury and fibroblast activation, providing a mechanistic rationale for its use in PM-associated fibrosis. Gambogic acid, a natural product with anti-inflammatory and antitumor properties, and butein, a flavonoid with antioxidant effects, offer complementary mechanisms for targeting HSPA5-driven pathways.

In conclusion, this study provides robust evidence for PM→IPF causality, identifies novel mediators/pathways, and proposes drug repositioning candidates. Our findings advance understanding of autoimmune-fibrotic disease links and offer new directions for targeted IPF therapies in PM patients.

## 5. Strengths and limitations

Our study has several strengths, including the application of a 2-sample MR framework to minimize confounding, a multi-omics mediation design to uncover biological pathways, and the use of large-scale GWAS data for robust genetic instruments.

However, this study has limitations. The MR methodology relies on key assumptions, and residual horizontal pleiotropy remains a potential concern despite sensitivity analyses. The use of public GWAS data introduces possible sample overlap, necessitating future validation in independent cohorts. The predominantly European ancestry limits generalizability, and the lack of preregistration designates this as an exploratory analysis. Finally, both the identified mediators and the drug candidates from preliminary in silico docking require experimental validation in physiological contexts to confirm their biological and therapeutic relevance.

## Acknowledgments

The authors would like to thank the FinnGen database and the public GWAS catalog for providing the data used in this study.

## Author contributions

**Data curation:** Miaomiao Chen, Ruiying Zhang, Yazheng Zhang.

**Formal analysis:** Xia Tong, Ruiying Zhang.

**Funding acquisition:** Miaomiao Chen, Bin Liu.

**Project administration:** Xin Zhang, Bin Liu.

**Resources:** Xia Tong.

**Software:** Xia Tong.

**Supervision:** Xia Tong.

**Writing** – **original draft:** Miaomiao Chen.

**Writing** – **review & editing:** Miaomiao Chen, Xin Zhang.














